# Cultural and linguistic validation of the NHQ-2 Questionnaire: a specific instrument for assessing patient’s usability of inhalation devices

**DOI:** 10.1186/s40248-016-0067-y

**Published:** 2016-08-23

**Authors:** Roberto W. Dal Negro, Massimiliano Povero

**Affiliations:** 1 National Centre for Respiratory Pharmacoeconomics & Pharmacoepidemiology - CESFAR, Verona, Italy; 2 AdRes Health Economics and Outcome Research, Torino, Italy

**Keywords:** Inhalation devices, New Handling Questionnaire-NHQ-2, Questionnaire validation, Patients’ preference, Usability

## Abstract

**Background:**

An increasing number of inhalation devices are presently available in the market. They are differently characterized in terms of their handling and usability, both factors which may affect the outcomes of respiratory treatment.

The assessment of the preference and the usability rate of all devices can be carried out by means of specific questionnaires. Before their use, the identification of errors due to the incorrect wording of questions included in the questionnaires, together with the trans-cultural reliability represents the main issues of their cultural and linguistic validation.

**Methods and results:**

The New Handling Questionnaire - NHQ-2 is a novel specific questionnaire aimed to measure both the preference and the usability of all kinds of inhalation devices. The method used for its validation has been summarized in the first section of the present paper, while the results of the specific validation and translation process have been described in the second section, together with the grading of improvement achieved over the process. The comprehensibility and the reproducibility rates achieved for both the Italian and the English final versions of the NHQ-2 questionnaire were very high, such as >90 % for each question included.

**Conclusions:**

The novel NHQ-2 questionnaire proved very high comprehensibility and reproducibility in both its Italian and English final versions. It can be proposed for the trans-cultural clinical use when the usability, and not only the patients’ preference of devices, should be assessed.

**Electronic supplementary material:**

The online version of this article (doi:10.1186/s40248-016-0067-y) contains supplementary material, which is available to authorized users.

## Background and Results

Respiratory drugs are most effectively delivered via the inhalation route, particularly in conditions characterized by the presence of airflow limitation (namely in bronchial asthma and in chronic obstructive pulmonary disease - COPD) because they target directly the lungs, offer a more rapid onset of action, allow the use of smaller doses, and consent a better efficacy-to-safety ratio compared to systemic treatments [1, 2].

Independently of the different drug(s) used, several aspects specifically related to inhalation devices can contribute to the effectiveness of respiratory treatments, and some of them are strictly related to technical aspects (such as: the capability to consent the inhalation of a sufficient respirable fraction of the drug, the dose reproducibility, the dose precision, the dose stability), but others are linked to their acceptability, preference, and usability by respiratory patients.

A huge number of different inhalation devices are presently available in the market, even if differently characterized in terms of their handling and usability. Despite both these aspects are absolutely critical for the choice of the most convenient device, only a few specific and validated research instruments are available in real life [3–7].

The questionnaire is the cheapest and the most widely used instrument for investigational purposes in this field of research, because, when reliable and validated also from the linguistic point of view, it allows to collect significant information on specific groups of individuals in short time [8–11].

The present paper will first shortly describe the essential methodological steps for both the *testing* and the *translation* of any questionnaire to validate [12–16]. In a second phase, these procedures will be applied to a specific questionnaire, such as the New Handling Questionnaire (NHQ-2), which is a novel tool aimed to assess the usability and preference of inhalation devices.

## Methods

### Essential methodological steps

The *pre-test* procedure represents the true crucial step in the process of developing a new questionnaire. Usually, the real effectiveness of this instrument is assessed by administering the original list of questions to a limited sample of subjects (usually people who are easily and quickly available, of both genders, with different cultural levels, and not necessarily participating in the real survey) during the *pre-test* phase [9–16]. Actually, a substantial proportion of mistakes are only identified thanks to the contribution of these potential respondents when they face the list of questions for the first time [16–21].

All questions included in the questionnaire should be checked in terms of their *validity and reliability*. The former one identifies the instrument’s ability to effectively measure one aspect with an accepted degree of precision, while the latter consists in its ability to provide reproducible measurements of the same topic, in similar conditions, over time [13, 14, 18–21].

When producing an effective investigational instrument in two languages, it is much more convenient to consider the two versions of the questionnaire [15, 22, 23]. One of the most widely used methods for “translating” a questionnaire from one language to another is the “translating/back-translating method”, which consists of four different operational phases [22, 23]: 1) the initial translation from the research language to the foreign language by a bilingual translator; 2) *back-translation:* in other words, a new translation, this time from the foreign language to the research language by a translator different from that one who performed the first translation, and who is not familiar with the original version of the questionnaire; 3) comparison between the two versions of the questionnaire written in the research language; 4) in the event of substantial differences between the two versions, the preparation of a new draft translation containing the necessary changes is needed. This procedure was strictly adopted for translating the NHQ-2 from Italian into English language.

The essential methodological steps for a questionnaire validation are summarized in Table 1.

**Table 1:** Methodological steps for a questionnaire validation in two languages

Pre-test	
administering the original list of questions to a limited, unselected sample of subjects;	
identification of mistakes;	
administering the revised list of questions;	
Validation	
check of each question’s validity (precision) and reliability (reproducibility);	
Translation of the definitive version of questions	
initial translation from the research language to the foreign language by a bilingual translator;	
back-translation: a new translation, from the foreign language to the research language by a different translator;	
comparison between the two translated versions of the questionnaire;	
proceed in order to obtain two corresponding versions, equally understandable, and a comprehension rate >90 % for each question.	

### The operational steps towards the definitive version of the novel questionnaire

The *NHQ-2* questionnaire deeply involves both patients and nurses. For this reason, according to the accepted validation procedures, the original Italian version of the questionnaire was firstly submitted to a sample of 26 individuals (the usual sample size for a pilot test) not educated in respirology (whose gender, age, and educational level are reported in Table 2), and to a limited sample of 6 expert respiratory nurses (4 females, aged 29–41 years). As previously claimed, the aim in this phase was to detect any structural or conceptual bias within the questionnaire, and/or the existence of linguistic errors and/or mis-understandings which should affect the effectiveness of questions.

**Table 2:** Characteristics of the sample of subjects involved in the pre-test phase

		Sex				Total
		Male		Female		
Education		High school or degree	Middle school	High school or degree	Middle school	
Age	<19	1	1	2	1	5
	19–60	3	4	4	3	14
	>60	2	1	2	2	7
Total		6	6	8	6	26

Possible devices to compare by means of the NHQ-2 questionnaire during a single session were limited to a maximum of four, belonging to any family of inhalation devices, and they are generically indicated as A, B, C, and D.

All questions had been written in a single line in order to facilitate both their reading and comprehension. The great majority of questions were closed questions, except the age, those concerning possible previous education providers, and those of items 1.a; 1.c.1; 2.b, and 2.d within the Assessing Track.

Subject were required to indicate (by checking with an X), or to describe their choice in the appropriate spaces: this procedure was clearly reported on the top of the questionnaire, in the second line, just under the name of the questionnaire.

Information concerning any previous experience with different families of inhalation devices are directly required to patients. In the section of the Assessing Track, all items in group 1 and 5, items 2.a; 2b in group 2 are involving the patients directly, differently from all other remaining items which involve the nurse only.

The NHQ-2 Questionnaire did not show any structural flaw in its original version; nonetheless some changes and corrections were needed. Firstly, a much more appropriate numbering system was introduced for some items (namely, items # 1; 2; 3, and 5), in order to clarify to respondents that each battery of these questions was dependent of the main previous comprehensive item. As some items are specifically addressed to patients, while other items to the nurse, whom the item is addressed to was clearly reported for all items.

The most difficult item in terms of comprehension was item # 2, because it consists of two sub-items addressed to patients (2.a and 2.b), and the remaining two to the nurse (2.c and 2.d), exclusively.

Moreover, some English terms had been explained and implemented in the Italian version. Namely, the original terms “DPI” (Dry Powder Inhaler); “MDI” (Metered Dose Inhaler), and “SMI” (Soft Mist Inhaler), well known to professionals, were scarcely known by patients or general population, and then they were implemented with the following descriptions, namely: “*Inalatore per polveri secche*”; “*Aerosol predosato*”, and “*Erogatore per nebbia inalatoria*”, respectively, because otherwise not understood by the great majority of subjects ([26.9, 53.8, and 19.2 %, respectively].

Moreover, also the term “*nurse”*, which was reported several times in the first page of the original version of NHQ-2 (see Assessing Track and Item # 1), was changed. Even if this term is actually commonly used also in Italian, it was effectively understood by only 34.6 % of subjects (9/26), likely due to the heterogeneity of the testing sample in terms of age and education level. The same fate had the term “*device”*, which was also repeated several times in all the items of the original Italian version of the questionnaire. These two terms were replaced with their Italian equivalents, namely “*tecnico/a”* and “*erogatore”,* respectively.

Questions corresponding to items 1.b; 1.c, and 1.c.1 of the validated version were reworded because they resulted mis-leading in their original version, and only a poor proportion of patients perceived their meaning properly (such as: 80.7 % (21/26); 76.9 % (20/26), and 73.13 % (19/26), respectively).

Moreover, the term “*in ordine crescente*” has been changed into “*da meno a più*” in the Italian version, because the original wording had been mis-understood by 61.5 % (16/26) of subjects. In particular, in items 2.a and 2.c the grading sequence of devices was facilitated by using ordinal numbers. This new formulation tended to minimize the possibility that the respondent may attribute the opposite meaning to his own response.

Other minor changes were made to the original version of the questionnaire. The items concerning the age, the sex, and the education degree of patients were shifted to the final section of the questionnaire. As they deal with personal details, this kind of questions might be perceived by subjects as irrespective of their privacy [8, 9].

Moreover, in order to emphasize the value of the patient role, a short thanking sentence was included at the end of the questionnaire.

The English version of the tested questionnaire was carried out by using the technique of translation and back-translation, with the contribution of human science professionals. The English translation of the original researcher’s version of the Handling Questionnaire and both the final validated versions in Italian and in English are reported in the Additional files 1 and 2, together with the corresponding percent rate of comprehension achieved for each item.

In order to better perceive and compare the different validation steps and their relevance in terms of comprehension, the *New Handling Questionnaire (NHQ-2)* was reported in Additional file 3 in its original Italian version, together with the percent rate of comprehension for each question measured at the first reading of the questionnaire. Finally, the definitive Italian and English versions of the questionnaire were reported in Additional files 1 and 2, together with their corresponding comprehensibility rates.

## Discussion

A questionnaire represents the most widespread, simple, cheap, helpful, and sometimes specific investigational instrument widely used for research concerning people’s behaviours and beliefs. The validity of a questionnaire in terms of full comprehension of all questions included, and of repeatability of the respondents’ responses still represents a crucial issue.

The procedures for assessing the reliability and the effectiveness of a given questionnaire are essential and mandatory in the validation process, because they allow the identification and the elimination of those mistakes and mis-understandings which could, otherwise, cause bias in the  final results of any survey. Actually, only once validated from these points of view, a novel questionnaire can be extensively used for its original purposes.

It is the case of the NHQ-2 Questionnaire, which always achieved a comprehension rate >90 % for each item included in both its final Italian and English versions.

When the domains covered by the NHQ-2 questionnaire are compared to those of other questionnaires, some relevant differences become easily clear. In particular, the previous Handling Questionnaire was only dedicated to Dry Powder Inhalers [6], while the NHQ-2 questionnaire represents a novel investigational tool aimed to mainly check and compare the overall usability of all pre-dosed inhalation devices (such as: Metered Dose Inhalers – MDI; Dry Powder Inhalers – DPI; Soft Mist Inhalers - SMI) independently of the drug(s) used, and not only the degree of patients’ preference.

Moreover, the concept of the NHQ-2 questionnaire is also different from that of the FSI-10 Questionnaire [7] which is a self-completed questionnaire for mainly assessing the patients’ satisfaction, but it is not provided with the objective control of expert nurses checking all the inhalation steps up to the first proper actuation of patients.

The NHQ-2 questionnaire was designed to be more oriented to the controlled assessment of patients’ practical skills in handling devices effectively, rather than to merely collect the common patients’ preference opinions, which can be biased by emotional factors. In other words, the NHQ-2 questionnaire was designed with the aim to better contribute to the true identification of those aspects which may critically limit patients’ usability of inhalation devices in real life, just stimulating their effective empowerment.

It is our opinion that the adding value of the NHQ-2 questionnaire stems from the higher critical value recognised to the operational role of the expert nurses who have to supervise, judge, and assess the reliability of all patients’ actions and responses required by the questionnaire, carefully and objectively.

## Conclusions

The novel NHQ-2 questionnaire proved a very high comprehensibility and reproducibility in both its Italian and English final versions. As its domain also widely covers the usability assessment of inhalation devices, it is proposed for the trans-cultural clinical use when the usability, and not only the patients’ preference of devices, should be assessed.

## Abbreviations

DPI, dry powder inhalers; MDI, metered dose inhalers; NHQ-2, the new handling questionnaire; SMI, soft mist inhalers

## Additional files

Additional file 1: Validated version in Italian language and the corresponding % rate of comprehension Questionario NHQ-2. (DOCX 35.1 kb) Additional file 2: Validated version in English language and the corresponding % rate of comprehension. (DOCX 33 kb) Additional file 3: The original version of the NHQ-2 Questionnaire together to the % rate of comprehension at the first reading Questionario – NHQ-2. (DOCX 41.6 kb)
