# Supplementary material for: Cultural and linguistic validation of the NHQ-2 Questionnaire: a specific instrument for assessing patient’s usability of inhalation devices
Source: Multidiscip Respir Med. 2016 Aug 23;11(1):32. doi: 10.1186/s40248-016-0067-y (PMC4994229; doi:10.1186/s40248-016-0067-y)
Supplement: Additional file 1: — Validated version in Italian language and the corresponding % rate of comprehension Questionario NHQ-2. (DOCX 35.1 kb) [file 40248_2016_67_MOESM1_ESM.docx]

**Appendix 1** **The original version of the NHQ-2 Questionnaire together to the % rate of comprehension at the first reading**

**Questionario – NHQ-2**

n.: ………

età ………… **Sesso**: M F

**Scolarità**: 0 = nessuna; 1 = elementare; 2 = media inf.; 3 = media sup.; 4 = laurea

Precedente istruzione all’uso del DPI? Si No se Si, da chi? .......................

*(7/26 – 26.9%)*

Precedente istruzione all’uso del MDI? Si No se Si, da chi?.......................

*(14/26 – 53.8%)*

Precedente istruzione all’uso del SMI? Si No se Si, da chi?.......................

*(5/26 -19.2%)*

**PERCORSO VALUTATIVO**

**A B C D**

**Durata della dimostrazione del nurse (in sec.): ...... ...... ..... .....**

*(6/6 – 100.0)*

1. **Dopo la spiegazione da parte del nurse:** *(9/26 – 34.9%)*

Quale device preferisce ? *(25/26 – 96.1%)*  **A B C D**

Quale device ritiene sia il più facile da usare ? *(21/26 – 80.7%)* **A B C**  **D**

La sua maggior difficoltà è con il device ?: *(20/26 – 76.9%)* **A B C**  **D**

dovuta a: *……………(19/26 – 73.1%)..………………………………………*

1. **Dopo che il paziente ha utilizzato personalmente il device:**

- **Ordini gli erogatori in ordine crescente in base alla difficoltà incontrata:** *(16/26 – 61.5%)*

**1° …… 2° …… 3° …… 4° ……..**

- **Indichi la maggior criticità incontrata con ogni device:** *(20/26 – 76.9%)*

**1 …………………… 2…………………… 3………………….. 4……………………..**

- **Ordini gli erogatori in base alla difficoltà dimostrata dal paziente:** *(5/6 -83.3%)*

**1° …… 2° …… 3° …… 4° ……..**

- **Indichi la maggior criticità incontrata dal paziente con ogni device:** *(6/6 – 100%)*

**1 …………………… 2…………………… 3………………….. 4……………………..**

1. **Numero di tentativi per la 1a corretta attuazione con ogni device:** *(6/6 – 100%)*

**A ……. B ……. C ……. D……..**

1. **Tempo totale per la 1a corretta attuazione con ogni device (in sec.)**: *(5/6 – 83.3%)*

**A …… B …… C …… D ……..**

1. **Indichi quale device preferisce in termini di:** *(25/26 - 96.0%)*

**Device ------------------------------------**

1. **Aspetto** *(26/26 – 100.0%)* **A B C D**
2. **Ingombro** *(26/26 – 100.0%)* **A B C D**
3. **Boccaglio** *(26/26 – 100.0%)* **A B C D**
4. **Igiene** *(25/26 – 96.1%)* **A B C D**
5. **Contatore di dosi** *(26/26 – 100.0%)* **A B C D**
6. **Impugnatura** *(26/26 – 96.1%)* **A B C D**
7. **Numero manovre** *(20/26 – 76.9%)* **A B C D**
8. **Facilità d’uso** *(26/26 – 100.0%)* **A B C D**
9. **Percezione dell’inalazione** *(26/26 – 100.0%)* **A B C D**
10. **Valvola di inalazione** *(20/26 – 76.9%)* **A B C D**
