# Supplementary material for: Cultural and linguistic validation of the NHQ-2 Questionnaire: a specific instrument for assessing patient’s usability of inhalation devices
Source: Multidiscip Respir Med. 2016 Aug 23;11(1):32. doi: 10.1186/s40248-016-0067-y (PMC4994229; doi:10.1186/s40248-016-0067-y)
Supplement: Additional file 3: — The original version of the NHQ-2 Questionnaire together to the % rate of comprehension at the first reading Questionario – NHQ-2. (DOCX 41.6 kb) [file 40248_2016_67_MOESM3_ESM.docx]

**Appendix 3 - Validated version in English language and the corresponding % rate of comprehension**

**The NHQ-2 Questionnaire**

(Check with an X, or describe your answer)

n.: ………

Previous instructions for the DPI use? Yes No if YES, from? ...................

(Dry powder Inhalers) *(25/26 – 96.1%)*

Previous instructions for the MDI use? Yes No if YES, from? ...................

(Metered Dose Inhalers) *(26/26 – 100.0%)*

Previous instructions for the SMI use? Yes No if YES, from? ..................

(Soft mist Inhalers) *25/26 – 96.1%)*

**ASSESSING TRACK**

**To the Nurse: please report the duration of your explanation (in sec.): A ...... B ......C ..... D .....**

*(6/6 – 100.0 %)*

1. **Questions to the patient, after the nurse’s explanation:**

1.a which device do you prefer “at glance”? *(26/26 – 100.0%)*  **A B C D**

1.b which device did you perceive as the easiest to use? (26/26 – 100.0%)  **A B C**  **D**

1.c which device did you perceive as the most difficult to use? *(25/26 – 96.1%)* **A B C**  **D**

1.c.1 why ?: *……………(26/26 – 100.0 %)………………………………………..*

1. **Questions to the patient and to the Nurse, After the patient’s practicing:**

2-a Please, grade devices by difficulties you encountered in their use (in increasing order):

**(PATIENT’S OPINION)** *(25/26 – 96.1%)*

**1st …… 2^nd^ …… 3rd …… 4th ……..**

2.b Please, report your most difficult step in actuation of each device:

(**PATIENT’S OPINION**) *(25/26 – 96.1%)*

**A …………………… B…………………… C………………….. D……………………..**

2.c Please, grade devices by difficulties the patient encountered (in increasing order):

(**NURSE’S ASSESSMENT**) *(6/6 – 100.0%)*

**1st …… 2^nd^ …… 3rd …… 4th ……..**

2.d Please, report the most difficult patient’s step in actuation of each device::

**(NURSE’S ASSESSMENT**) *(6/6 – 100.0%)*

**A …………………… B…………………… C………………….. D……………………..**

1. **Please, report the overall n. attempts for the 1^st^ proper actuation with each device:**

**(NURSE’S ASSESSMENT)** *(6/6 – 100.0%)*

**A …… B …… C …… D……..**

1. **Total time spent for the 1^st^ proper actuation with each device (in sec.)**

**(NURSE’S ASSESSMENT)** *(6/6 – 100.0%)*

**A …… B …… C …… D ……..**

1. **As, each device shows some different structural features,**

**please indicate which device you prefer in terms of :**

**(Patient’s Preference)** *(26/26 – 100.0%)*

**Device ------------------------------------**

1. **Shape** *(26/26 – 100.0%)* **A B C D**
   1. **Size** *(26/26 – 100.0%)* **A B C D**
2. **Mouthpiece** *(26/26 – 100.0%)* **A B C D**
3. **Hygiene** *(26/26 – 100.0%)* **A B C D**
4. **Dose counter** *(26/26 – 100.0%)* **A B C D**
5. **Ease of gripping** *(26/26 – 100.0%)* **A B C D**
6. **N. actuation manoeuvres** *(25/26 – 96.1%)* **A B C D**

1. **Ease of use** *(26/26 – 100.0%)* **A B C D**
2. **Perception of inhaled dose** *(26/26 – 100.0%)* **A B C D**
3. **Trigger valve** *(25/26 – 96.1%)* **A B C D**

**Age** …………

**Gender** M F

**Education**: 0 = none; 1 = elementary; 2 = middle; 3 = high; 4 = degree

**Thank you for your valuable cooperation**
